# The Influence of Aerobic Exercise Therapy on Patient-Reported Outcome Measures Following Concussion: A Scoping Review

**DOI:** 10.3390/biomedicines13112615

**Published:** 2025-10-25

**Authors:** Dean M. Cordingley, Serena C. L. Buchwald, Izabella Marquez, Frederick A. Zeiler

**Affiliations:** 1 Pan Am Clinic Foundation, Winnipeg, MB R3M 3E4, Canada; sbuchwald@panamclinic.com (S.C.L.B.); frederick.zeiler@umanitoba.ca (F.A.Z.); 2 Applied Health Sciences Program, Faculty of Graduate Studies, University of Manitoba, Winnipeg, MB R3T 2N2, Canada; 3 Department of Biosystems Engineering, Price Faculty of Engineering, University of Manitoba, Winnipeg, MB R3T 5V6, Canada; 4 Department of Biomedical Engineering, Price Faculty of Engineering, University of Manitoba, Winnipeg, MB R3T 5V6, Canada; 5 Section of Neurosurgery, Department of Surgery, Rady Faculty of Health Sciences, University of Manitoba, Winnipeg, MB R3A 1R9, Canada; 6 Centre on Aging, University of Manitoba, Winnipeg, MB R3T 2N2, Canada; 7 Division of Anaesthesia, Department of Medicine, Addenbrooke’s Hospital, University of Cambridge, Cambridge CB2 0AW, UK; 8 Department of Clinical Neuroscience, Karolinska Institutet, 171 77 Stockholm, Sweden

**Keywords:** mild traumatic brain injury, rehabilitation, quality of life, aerobic exercise

## Abstract

**Background/Objectives**: The purpose of this scoping review was to identify the effects of exercise therapy on patient-reported outcome measure(s) (PROM(s)) after concussion, and to highlight the current knowledge gaps in the literature. **Methods**: A search of EMBASE, MEDLINE, SCOPUS, BIOSIS, and Cochrane Libraries was performed on 1 December 2023 (from database inception) and updated on 19 June 2025 to review the available literature. The Preferred Reporting Items for Systematic Reviews and Meta-Analyses (PRISMA) extension for scoping reviews was used to report the scoping review. An online review management system was used to remove duplicates and perform article screening. **Results**: Following the removal of duplicates, 5434 articles were identified for screening. After applying the inclusion/exclusion criteria, 27 articles remained for data extraction. The available literature has found that applying structured aerobic exercise following concussion can improve patient-reported symptom severity compared with control groups. **Conclusions**: There is currently a paucity of research reporting on the impact of aerobic exercise therapy following concussion on PROMs aside from patient-reported symptom severity. Additional PROMs recommended as common data elements should be included in future studies investigating the effects of aerobic exercise therapy following concussion.

## 1. Introduction

Exercise therapy for the treatment of numerous health conditions and diseases is well recognized [1]. More recently, the concept that ‘Exercise is Medicine’ has expanded to the management of concussions [2]. Previously, the avoidance of exercise following a concussion was recommended, as it was suggested that exercise early after a concussion may increase the time to recovery [3,4] and put individuals at risk during a time when neuronal cells may be vulnerable to further injury [5,6]. Additionally, prolonged rest following a concussion may be associated with delayed return to school/work [7]. However, there are now many clinical trials, systematic reviews, and meta-analyses showing that exercise therapy (specifically sub-symptom threshold aerobic exercise) can improve time to medically determined recovery and increase the rate of symptom resolution.

Currently, the mechanistic actions of exercise therapy on the concussed brain are not fully elucidated [8,9]. Equally important, or possibly more important, is understanding the effect that exercise therapy has on patient-reported outcome measures. As previously mentioned, evidence indicates that sub-symptom threshold aerobic exercise therapy can improve patient-reported symptoms when initiated acutely after a concussion or in individuals with persisting post-concussive symptoms. However, patient-reported symptoms are just one component of the patient-reported outcome measures that are recommended as common data elements for concussion and mild traumatic brain injury [10,11]. Therefore, the purpose of this scoping review is to identify the effects of aerobic exercise on patient-reported outcomes and to identify the current gaps in the literature.

## 2. Materials and Methods

This scoping review followed the *Cochrane Handbook for Systematic Reviews* and the scoping review extension of the *Preferred Reporting Items for Systematic Reviews and Meta-Analyses* (PRISMA) [12]. Approval from the Institutional Research Ethics Board was not required for this scoping review. The protocol was registered with the Open Science Framework (https://osf.io/2dcb5/. Accessed on 18 September 2025).

### 2.1. Research Question

The research question was as follows: “What is the effect of structured aerobic exercise therapy on patient-reported outcomes following concussion?”

For this review, a healthcare professional needed to diagnose the individual as having a concussion, and the patient needed a Glasgow coma scale (GCS) score ≥ 13. For studies that did not note patient GCS, descriptive information on symptom severity and any limitations in daily activities, etc., were identified, and author judgment was used.

Any mode of exercise (i.e., cycle ergometer, treadmill, track, etc.) that was at a submaximal intensity was included as aerobic exercise.

All outcomes directly reported by the patients relating to their symptoms, quality of life, health status, or functional status were included as PROMs. Appendix A contains a full list of search terms.

### 2.2. Search Strategy

On 1 December 2023, EMBASE, MEDLINE, SCOPUS, BIOSIS, and Cochrane Libraries were searched for relevant literature, with an updated search performed on 19 June 2025 to ensure currency. The databases were searched back to inception, and reference sections for included papers were also searched for additional literature. Two researchers who were topic experts and had experience designing and conducting scoping reviews developed the search strategy (DMC and FAZ). The removal of duplicates and article screening were performed using an online software for scoping review management (Rayyan, Cambridge, MA United States of America. https://www.rayyan.ai) by three researchers (DMC, SCLB, and IM). A sample search strategy is available in Appendix A, and the PRISMA checklist is available in Appenidx B.

### 2.3. Eligibility Criteria

#### 2.3.1. Inclusion Criteria

Articles must have been published in peer-reviewed journals and needed to have an aerobic exercise intervention initiated after a concussion or mild TBI to be included. Human studies with participants of any age were included. The participants must have been diagnosed with a concussion or mild TBI without any reported polytrauma.

#### 2.3.2. Exclusion Criteria

All conference abstracts/papers, case reports, case series, or literature that were not original research were excluded. Studies that incorporated multiple interventions or did not involve an aerobic exercise intervention were excluded. All studies that utilized an animal model were excluded. Additionally, studies that did not measure any PROMs, were non-English, or included moderate and/or severe TBI populations where it was not possible to discern outcomes specific to concussion/mild TBI population were excluded.

### 2.4. Article Selection Process

For the screening process, titles and abstracts were initially screened by two researchers (DMC and SCLB), and all identified full-texts were reviewed by the same researchers. In the case of a disagreement, a third researcher (FAZ) was included to reach consensus.

### 2.5. Data Charting Process

All full-text articles that met the inclusion and exclusion criteria had their data extracted (see Table A2 and Table A3). Each study had data extracted to identify the study design, characteristics of the study participants, general study methodologies, detailed description of the aerobic exercise intervention, all study outcome measures, detailed PROMs collected in relation to the aerobic exercise intervention, study results specific to the PROMs, and study conclusions.

### 2.6. Data Items

The extracted data included patient characteristics (including sample size, biological sex, and age), study design, exercise intervention, outcome measures, study results, and general conclusions of the study.

### 2.7. Bias Assessment

A formal bias assessment was not conducted, as the purpose of this scoping review was to provide an overview of the current literature investigating the influence of an aerobic exercise intervention on PROMs following mild TBI/concussion. However, few of the studies were primary randomized clinical trials [13,14,15,16,17], with many studies at risk of being inappropriately powered due to being pilot studies [18,19,20,21,22,23,24,25,26,27,28] or secondary analyses [29,30,31,32,33]. The remaining study designs included cohort [34,35], case series [36], and quasi-experimental studies [37,38], which all have their own associated risks of bias.

### 2.8. Synthesis of Results

No statistical analysis was performed. The extracted data were described to outline their results and an overview was provided.

## 3. Results

### 3.1. Search Results and Study Characteristics

**Figure 1:**
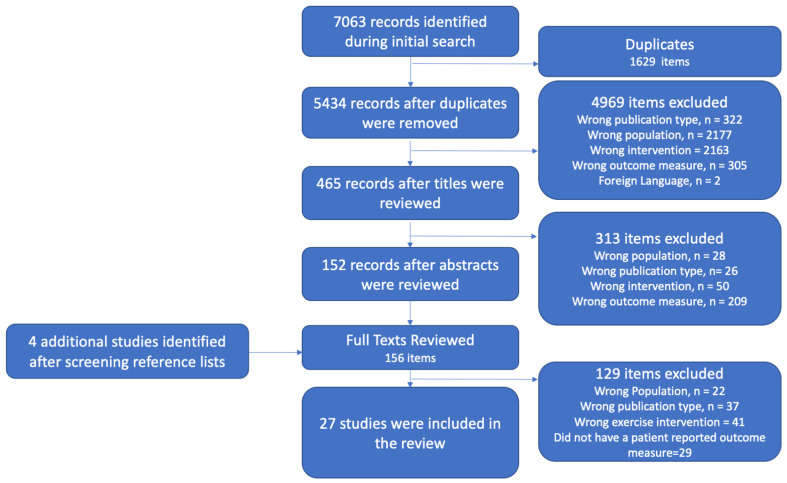
PRISMA flow diagram.

A total of 7063 articles were identified from all included databases upon the initial search. There were 1629 duplicates identified and deleted, leaving 5434 studies for title screening. Title screening resulted in 4969 articles being identified as not relevant. The abstracts of 465 articles were reviewed, and a total of 152 articles remained for full-text screening. Full-text screening resulted in the removal of 128 articles, leaving 24 studies to be included. Reviewing the references of the studies included resulted in four additional studies being identified for full-text review, three of which met the inclusion/exclusion criteria. Implementation of the search strategy and our inclusion/exclusion criteria resulted in a total of 27 studies being included for this review (see Figure 1 for the PRISMA flow diagram).

**Figure 2:**
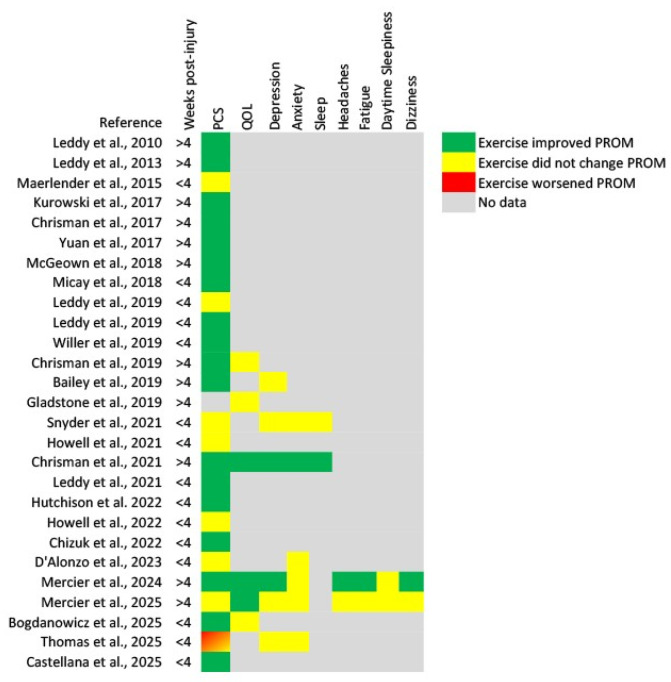
Heat map of results for each patient-reported outcome measure from each identified study [13,14,15,16,17,18,19,20,21,22,23,24,25,26,27,28,29,30,31,32,33,34,35,36,37,38,39]. PCS, post-concussion symptoms; QOL, quality of life.

The majority of identified studies included both men and women participants [13,14,15,16,17,19,21,22,23,24,25,26,27,28,29,30,31,32,33,35,36,37,39], while only two studies included only men [20,38]. There were 12 studies that included participants who were more than four weeks post-concussion [14,19,22,23,24,25,27,28,33,35,36,39], and 16 studies that included participants who were enrolled less than four weeks post-concussion [13,15,16,17,18,20,21,26,29,30,31,32,34,37,38]. Additionally, varying prescribed exercise interventions were utilized between studies. The exercise interventions were prescribed based on perceived exertion [13,26], target heart rate [19], a target heart rate based on a percentage of the participant’s age-predicted maximum [16,20,21,28], or individualized based on the participants performance during an exercise tolerance test [14,15,17,18,22,23,24,25,27,29,30,31,33,34,35,36,37,38,39]. Patient-reported symptom severity was consistently reported throughout studies; other PROMs evaluated explored patient quality of life, anxiety and depression, and sleep. Relevant PROMs and study characteristics are listed in Table A2, while PROMs’ responses to an aerobic exercise intervention following mild TBI/concussion are noted in Table A3. A summary of the results for each PROM is found in Table 1, and a summary for each study is found in Figure 2.

### 3.2. Symptom Severity Response to Aerobic Exercise Therapy

The most common PROM evaluated in the included studies was symptom severity. The earliest study by Leddy et al. (2010) was conducted in a cohort of adolescent and adult patients with persistent post-concussion symptoms between 6 and 40 weeks post-injury [36]. It was demonstrated that sub-symptom threshold aerobic exercise results in decreased symptom severity and improved rates of symptom recovery when typical recovery, as assessed during the ‘baseline’ phase, is controlled for [36]. A follow-up study that added a placebo group (assigned a stretching protocol) evaluated the same sub-symptom threshold aerobic exercise program in a group of adults with persistent concussion symptoms [24]. Interestingly, they found that the group who performed the sub-symptom threshold aerobic exercise improved their number of symptoms (*p* < 0.0004), while the placebo group did not change (*p* = 0.16). These findings have since been corroborated, establishing the efficacy of aerobic exercise therapy in improving patient-reported symptoms in individuals with persistent concussion symptoms [19,22,23,25,27,28,35,39]. However, interventions need to be of sufficient duration, as a six-week aerobic intervention in adults with persisting post-concussion symptoms did not result in improved symptom burden compared to a stretching protocol [14].

Studies have also investigated aerobic exercise therapy in patients who had symptoms for <4 weeks. The earliest identified study by Maerlender et al. (2015) found no difference in the time it took for adolescent concussion patients to be symptom-free between a mild-to-moderate exercise group and a standard care group [26]. More recently, additional studies have supported the finding that an individualized aerobic exercise program may not improve patient-reported symptoms compared to standard care [13,18,21,31,34]. However, other studies have found that aerobic exercise therapy improves the rate of symptom resolution compared to placebo (passive stretching is commonly utilized) or standard care [15,16,17,20,37,38]. Additionally, early aerobic exercise therapy after a concussion may reduce the risk of adolescents developing PPCS [29]. One possible factor that may contribute to these contrasting findings is participant compliance, where participants who are more compliant and complete a higher volume of their prescribed aerobic exercise intervention report greater improvements in symptom severity compared to those who are not compliant [32,34].

### 3.3. Changes in Patient-Reported Quality of Life with Aerobic Exercise Therapy

The search resulted in six studies that evaluated patient-reported quality of life over time and in response to aerobic exercise therapy in individuals with persistent concussion symptoms [14,19,23,30,33,35]. These studies suggest that participants who perform aerobic exercise report an improved health-related quality of life, but to no different extent than individuals who do not receive aerobic exercise therapy [23,30,33] or receive delayed aerobic exercise therapy [35]. However, one study by Mercier et al. (2025) found that a six-week aerobic exercise therapy program resulted in improved quality of life compared to a stretching protocol with per-protocol analysis, but not with intention-to-treat analysis [14]. However, the mean change of 7.72 points on the Quality Of Life After Brain Injury scale (QOLIBRI) with aerobic exercise therapy [14] did not reach the minimally important clinical difference of 12 points previously identified following brain injury [40]. This suggests that patient compliance may contribute to the previously observed outcomes.

### 3.4. Changes in Patient-Reported Depression and Anxiety with Aerobic Exercise Therapy

Several of the identified studies investigated patient-reported depressive symptoms as an outcome measure. Over a six-week intervention period in individuals with persistent concussion symptoms, depression improves throughout the recovery of a concussion, but it is unclear if it is influenced by aerobic exercise compared to standard care [13,14,19,22] or delayed aerobic exercise [35]. Shorter durations of intervention (i.e., seven days) do not result in improved reporting of depression in either standard care or aerobic exercise interventions for individuals around 19 days post-injury [21].

Similar to patient-reported depression, few studies investigated patient-reported anxiety. The identified studies show that over a seven-day intervention period, symptoms of trait anxiety, but not state anxiety (evaluated with STAI), can improve in participants with concussions approximately 19 days post-injury [21], while a six-week intervention can improve symptoms of anxiety for individuals with persistent concussion symptoms [19], but possibly to no greater extent than stretching exercise [14]. The change in anxiety symptoms over seven days was no greater than standard care [21], and it is not known whether there is a difference compared to standard care over the longer six-week time period [19]. Additionally, aerobic exercise interventions implemented early after a concussion do not improve patient-reported anxiety to a greater extent compared to the placebo condition (stretching) [31], usual care [13], or delayed aerobic exercise [35].

### 3.5. Other Patient-Reported Outcomes Measured in Association with Aerobic Exercise Therapy

Several additional PROMs have been investigated, but are all limited to being included in only two studies each. The effects of aerobic exercise interventions on sleep have been investigated [19,21]. Based on the limited literature, over a six-week aerobic exercise intervention period, individuals with persistent concussion symptoms (~75 days post-injury) show improved sleep compared to baseline sleep quality [19]. However, the meaningfulness of the 7.67 point improvement on the Adolescent Sleep–Wake Scale (ASWS) is unknown [19], as an MCID for a concussion or TBI population could not be found. Additionally, a seven-day intervention is insufficient to alter sleep in individuals with more acute (~19 days post-injury) concussions, and there was no standard care or placebo condition, with no difference between individuals undergoing aerobic exercise therapy and those receiving standard care [21]. The functional impact of headache, fatigue, daytime sleepiness, and dizziness has been investigated in two studies by the same research group [14,35]. A six-week aerobic exercise intervention resulted in improved functional impact of headache and fatigue, but these improvements were not different from patients who completed a six-week stretching protocol [14]. Similarly, a 12-week aerobic exercise intervention resulted in improved impact of headache, fatigue, and dizziness [35]. However, Mercier et al. (2024) found that the immediate initiation of a 12-week aerobic exercise program improves the impact of headaches, fatigue, and dizziness to a greater extent than in individuals who delayed the aerobic exercise for six weeks [35].

## 4. Discussion

The purpose of this scoping review was to identify the effects of aerobic exercise on patient-reported outcomes and to identify the current gaps in the literature. This study identified a limited number of PROMs reported in association with prescribed exercise-based interventions following concussions. The most common PROM evaluated in the literature was a symptom severity score, with fewer studies reporting on patient-reported quality of life, anxiety, depression, and sleep quality. The available literature used a variety of methods to prescribe the exercise intervention, with varying durations, mixed-age patients, differences in time since injury, and different tools to evaluate the same PROMs. Additional research is needed to establish the role prescribed aerobic exercise could play in the response of PROMs.

Patient-reported symptom severity was the most frequently evaluated PROM [13,14,15,16,17,18,19,20,21,22,23,24,25,26,27,28,29,30,31,32,34,35,36,37,38,39]. The literature supports that an aerobic exercise intervention improves patient-reported symptoms in patients with persistent post-concussion symptoms (>4 weeks post-injury) [19,22,23,24,25,27,28,36,39]. However, the benefits of aerobic exercise therapy on patient-reported symptoms in patients <4 weeks post-injury are mixed, with some studies finding no benefit [13,18,21,26,31,34] and others finding beneficial outcomes compared to standard care or placebo [15,16,17,20,37,38]. One possible explanation for the differing findings could be compliance, where individuals who performed a greater volume of exercise and complied with their exercise prescription showed beneficial outcomes compared to individuals who did not comply with their prescription [14,32,34].

Compared to patient-reported symptom severity, there were far fewer studies that evaluated other PROMs. Patient-reported quality of life was evaluated in six studies and showed improvement over time, similar to standard care [14,19,23,30,33,35]. However, all but one of these studies [30] were conducted in individuals with persistent post-concussion symptoms for >4 weeks. Further studies on the effects of aerobic exercise initiated early post-concussion on patient-reported quality of life are needed. Also, additional studies that utilize comparable exercise interventions across different ages and time since injury are needed to draw a more complete picture of how aerobic exercise therapy may impact patient-reported quality of life.

Patient-reported depressive symptoms were evaluated in six studies [13,14,19,21,22,35], while patient-reported anxiety was examined in six studies [13,14,19,21,31,35] and sleep in two studies [19,21]. Due to the few number of studies and the use of differing patient populations, four studies were in patients with persistent post-concussion symptoms [14,19,22,35] and one in patients <4 weeks post-injury [21], it is not possible to draw conclusions on the effects of aerobic exercise therapy on patient-reported depression, anxiety, and sleep compared to standard care.

The results of this scoping review identified that few studies investigate PROMs in association with aerobic exercise-based therapy, and that patient-reported symptoms are the main measurement. Future research should evaluate an array of PROMs to develop a further understanding of the holistic patient. The inclusion of PROMs evaluating social aspects, sleep, quality of life, psychological, vestibular, neuromotor function, mood, global outcomes, and family and environmental outcomes post-concussion would provide a more inclusive understanding of the role aerobic exercise could play in a patient’s treatment plan.

One consideration not investigated in the identified literature is the potential role that genetic makeup could play in patient response to exercise therapy following concussion. An individual’s genetic composition could greatly impact their exercise performance and trainability [41,42,43]. Genetic variations may contribute to the heterogeneity of functional adaptations in older adults [44] and cardiovascular and metabolic adaptations to physical activity and exercise [43]. Therefore, certain individuals with a favorable genetic makeup may respond better to a given exercise therapy than others [43]. Symptomology and outcomes following concussion and mTBI are highly variable between individuals [45], which is possibly due to genetic factors [46]. Therefore, the influence of genetic makeup could greatly influence individual outcomes of exercise-based therapy following concussion.

### 4.1. Limitations of the Literature

The primary limitation of the literature is that most studies only investigated patient-reported symptom severity, and few studies investigated other PROMs. Additionally, the identified studies utilized different exercise interventions, including using different methods for prescribing exercise intensity, implemented the exercise intervention for different durations, and initiated the aerobic exercise at different time points post-concussion.

### 4.2. Limitations of This Review

A limitation of this review is that only studies published in English, or with an English translation available, were included in this scoping review due to the authors’ abilities. Also, it is not possible or appropriate to draw meaningful conclusions from this review for many of the assessed PROMs due to the limited volume of studies which assessed them, as well as the varying methodologies and demographics in the studies. Another limitation of the literature is that some studies did not report the specific patient-reported symptom severity and instead included the return of symptoms to baseline or reporting of no symptoms as a criterion for medical clearance. It was therefore assumed that, unless otherwise noted in the study, the patient’s medical clearance corresponded with the resolution of symptoms.

### 4.3. Future Directions

Future research aimed at understanding the effects of aerobic exercise therapy on PROMs beyond symptom severity is needed to fully understand the impact on the holistic patient. Also, future studies should investigate the influence of sex and gender on PROMs in relation to aerobic exercise therapy, since both sex and gender are known to sway injury presentation and recovery time [47,48,49,50]. Finally, in association with aerobic exercise therapy, future research should utilize serial collection of multiple data sources to try and elucidate the relationship between patient demographics, medical history, biological and physiological markers (i.e., biomarkers of injury and neurotrophic factors, cerebral physiologic measures, and quantified tractography and connectivity assessments with neuroimaging), and short- and long-term clinical outcomes and PROMs. Understanding the association of the numerous outcomes could assist in the development of personalized exercise treatment plans to best benefit specific patient populations. The development of a curated polygenic score repository would assist in understanding the genetic influence aerobic exercise may or may not have on an individual following a concussion [51,52].

## 5. Conclusions

This scoping review highlights the limited available literature that investigates the influence of aerobic exercise therapy on PROMs. Aerobic exercise therapy appears to be beneficial in improving patient-reported symptom severity in patients with persistent post-concussion symptoms and possibly beneficial in patients with more acute concussions as well. The literature investigating patient-reported quality of life, depression, anxiety, and sleep was too limited to draw conclusions due to the few studies, varying exercise interventions, and different initiation times of the interventions. Expanding our understanding of the effects of aerobic exercise therapy after concussion on a broader range of PROMs would assist in understanding the full scope of the role exercise therapy could play after brain injury.

## Figures and Tables

**Table 1:** Summary of results.

Outcome Measure	Assessment Tools	Results	Conclusion
Symptom Severity [13,14,15,16,17,18,19,20,21,22,23,24,25,26,27,28,29,30,31,32,34,35,36,37,38,39]	ImPACT, various versions of SCAT, PCSI, GSC, PCSS, HBI, PCS-R, RPQ	Aerobic exercise typically improves patient-reported symptoms in individuals with PPCS compared to placebo or standard care [19,22,23,24,25,27,28,35,36,39]. However, for a more acute concussion, some have found benefits of aerobic exercise treatment compared to placebo or standard care, [15,16,17,20,30,37,38] but others have found no difference [18,21,26,31,34]. One study suggests early aerobic exercise may result in worse symptoms early after a concussion (5 days post-injury) and may increase the time to recovery [13].	Aerobic exercise improves patient-reported symptoms for individuals with PPCS, but the findings are mixed for individuals with more acute concussions. Aerobic exercise does not worsen patient-reported symptoms in individuals early after their concussion, but it may not be more beneficial than standard treatment.
Quality of Life [14,19,23,30,33,35]	PedsQL, QOLIBRI	Patient-reported quality of life is not different for individuals treated with aerobic exercise compared to those provided with standard care. However, patient compliance to aerobic exercise may contribute to the lack of difference [14].	Aerobic exercise does not appear to improve patient-reported quality of life to a greater extent than standard care. However, the current literature is limited, making it not possible to draw definitive conclusions.
Depression [13,14,19,21,22,35]	BDI-2, PHQ-9, BSI-18	Throughout recovery, patient-reported depressive symptoms improve. However, it is currently unclear if aerobic exercise treatment influences the trajectory of improvement.	The current literature is limited and utilizes different study designs, making it not possible to draw conclusions.
Anxiety [13,14,19,21,31,35]	GAD-7, PROMIS Anxiety Short Form, STAI, BSI-18	Patient-reported anxiety improves over time, but it is unclear if aerobic exercise treatment results in a beneficial outcome.	The current literature is limited and utilizes different study designs, making it not possible to draw conclusions.
Sleep [19,21]	Sleep Problem Index-II, Adolescent Sleep–Wake Scale-10	Aerobic exercise treatment in individuals with PPCS may improve sleep quality, but it may not improve sleep over a shorter intervention period in individuals with more acute concussions.	There were only two identified studies that utilized different populations and different study designs, making it not possible to draw conclusions.
Functional Impact of Headaches [14,35]	HIT-6	The functional impact of headaches improves with aerobic exercise, but to a similar extent as standard care.	There were only two identified studies that utilized different study designs, making it not possible to draw conclusions.
Fatigue [14,35]	FSS	Fatigue following a concussion improves with aerobic exercise, but to a similar extent as standard care	There were only two identified studies that utilized different study designs, making it not possible to draw conclusions.
Daytime Sleepiness [14,35]	ESS	Daytime sleepiness is not altered with aerobic exercise treatment	There were only two identified studies that utilized different study designs, making it not possible to draw conclusions.
Dizziness [14,35]	DHI	It is not clear if dizziness following a concussion is modified with aerobic exercise treatment.	There were only two identified studies that utilized different study designs, making it not possible to draw conclusions.

ImPACT; SCAT, Sport Concussion Assessment Tool; PCSI, Post-Concussion Symptom Inventory; GSC, Graded Symptom Checklist; PCSS, Post-Concussion Symptom Scale; HBI, Health and Behavior Inventory; PCS-R, Post-Concussive Scale—Revised; PPCS, Persistent Post-Concussion Symptoms; PedsQL, Pediatric Quality of Life; BDI, Beck Depression Inventory; GAD-7, Generalized Anxiety Disorder Scale-7; PROMIS, Patient-Reported Outcomes Measurement Information System; STAI, State-Trait Anxiety Inventory; BSI-18, Brief Symptom Inventory-18; HIT-6, Headache Impact Test; FSS, Fatigue Severity Scale; ESS, Epworth Sleepiness Scale; DHI, Dizziness Handicap Inventory.

## Data Availability

No new data were created for the purpose of this study.

## Abbreviations

The following abbreviations are used in this manuscript:

GCS	Glasgow Coma Scale
PRISMA	Preferred Reporting Items for Systematic Reviews and Meta-Analyses
PROMs	Patient-Reported Outcome Measures
TBI	Traumatic Brain Injury

## Appendix A. Search Terms and Conditions

(Mild traumatic brain injury OR Mild TBI OR Brain injury OR Head injury OR Head trauma OR Cerebral trauma OR Brain trauma OR Cerebral Injury OR Concussion OR Closed brain injury OR Acute brain injury OR Brain injuries OR Cerebrovascular trauma OR Mild concussion OR Cerebral Concussion OR Brain Injuries, Traumatic) AND (Exercise OR Physical activit* OR Aerobic exercise OR Light intensity exercise OR Moderate intensity exercise OR Exercise training OR Acute exercise OR Leisure Activit* OR Recreation OR Exercise Therapy OR Rehabilitation Exercise* OR Exercise Therapies) AND (Post Concussion Syndrome*OR Post-Concussion Syndrome* OR Post-concussive Symptom* OR Post concussive Symptom*OR Post-concussion Symptom* OR Post concussion Symptom* OR Post-Concussion Symptom* OR Chronic post-concussive syndrome* OR Chronic post concussive syndrome* OR Function Recoveries OR Function Recovery OR Recovery of Function* OR Patient-Relevant Outcome* OR Patient Relevant Outcome* OR Clinical Effectiveness OR Treatment Effectiveness OR Rehabilitation Outcome* OR Treatment Outcome* OR Treatment Efficacy OR Clinical Efficacy OR Quality of life OR Life Quality OR Health-related quality of life OR Health Related Quality of Life OR HRQOL OR Cognition OR Cognitive function* OR Memory OR Mental Recall OR Retention OR Spatial Memory OR Short-term memory OR Long-term memory OR Return to Sport* OR Return to Play OR Return to Sporting Activit* OR Resumption of Sporting Activit* OR Sporting Activity Resumption* OR Activities Resumption* OR Return to Recreational Activit* OR Recreational Activities Resumption* OR Return to School* OR Return to Work)

## Appendix B. PRISMA-ScR Checklist

**Table A1:** Preferred Reporting Items for Systematic Reviews and Meta-Analyses extension for scoping reviews (PRISMA-ScR) Checklist.

SECTION	ITEM	PRISMA-ScR CHECKLIST ITEM	REPORTED ON PAGE #
TITLE			
Title	1	Identify the report as a scoping review.	1
ABSTRACT			
Structured summary	2	Provide a structured summary that includes the following (as applicable): background, objectives, eligibility criteria, sources of evidence, charting methods, results, and conclusions that relate to the review questions and objectives.	1
INTRODUCTION			
Rationale	3	Describe the rationale for the review in the context of what is already known. Explain why the review questions/objectives lend themselves to a scoping review approach.	2
Objectives	4	Provide an explicit statement of the questions and objectives being addressed with reference to their key elements (e.g., population or participants, concepts, and context) or other relevant key elements used to conceptualize the review questions and/or objectives.	2
METHODS			
Protocol and registration	5	Indicate whether a review protocol exists; state if and where it can be accessed (e.g., a web address); and if available, provide registration information, including the registration number.	3
Eligibility criteria	6	Specify characteristics of the sources of evidence used as eligibility criteria (e.g., years considered, language, and publication status) and provide a rationale.	3
Information sources *	7	Describe all information sources in the search (e.g., databases with dates of coverage and contact with authors to identify additional sources), as well as the date the most recent search was executed.	3
Search	8	Present the full electronic search strategy for at least 1 database, including any limits used, such that it could be repeated.	Appendix A
Selection of sources of evidence †	9	State the process for selecting sources of evidence (i.e., screening and eligibility) included in the scoping review.	3–4
Data charting process ‡	10	Describe the methods of charting data from the included sources of evidence (e.g., calibrated forms or forms that have been tested by the team before their use, and whether data charting was performed independently or in duplicate) and any processes for obtaining and confirming data from investigators.	4
Data items	11	List and define all variables for which data were sought and any assumptions and simplifications made.	4
Critical appraisal of individual sources of evidence §	12	If performed, provide a rationale for conducting a critical appraisal of included sources of evidence; describe the methods used and how this information was used in any data synthesis (if appropriate).	4
Synthesis of results	13	Describe the methods of handling and summarizing the data that were charted.	4
RESULTS			
Selection of sources of evidence	14	Give the number of sources of evidence screened, assessed for eligibility, and included in the review, with reasons for exclusions at each stage, ideally using a flow diagram.	4–5
Characteristics of sources of evidence	15	For each source of evidence, present characteristics for which data were charted and provide the citations.	5–8
Critical appraisal within sources of evidence	16	If performed, present data on critical appraisal of included sources of evidence (see item 12).	N/A
Results of individual sources of evidence	17	For each included source of evidence, present the relevant data that were charted that relate to the review questions and objectives.	Figure 2 and Appendix C Table A2 and Table A3
Synthesis of results	18	Summarize and/or present the charting results as they relate to the review questions and objectives.	Table 1 and Figure 2
DISCUSSION			
Summary of evidence	19	Summarize the main results (including an overview of concepts, themes, and types of evidence available), link to the review questions and objectives, and consider the relevance to key groups.	9–10
Limitations	20	Discuss the limitations of the scoping review process.	10
Conclusions	21	Provide a general interpretation of the results with respect to the review questions and objectives, as well as potential implications and/or next steps.	10–11
FUNDING			
Funding	22	Describe sources of funding for the included sources of evidence, as well as sources of funding for the scoping review. Describe the role of the funders in the scoping review.	11

PRISMA-ScR = Preferred Reporting Items for Systematic Reviews and Meta-Analyses extension for scoping reviews. * where sources of evidence (see second footnote) are compiled from, such as bibliographic databases, social media platforms, and websites. † a more inclusive/heterogeneous term used to account for the different types of evidence or data sources (e.g., quantitative and/or qualitative research, expert opinion, and policy documents) that may be eligible in a scoping review as opposed to only studies. ‡ the frameworks by Arksey and O’Malley (6) and Levac and colleagues (7) and the JBI guidance (4, 5) refer to the process of data extraction in a scoping review as data charting. § the process of systematically examining research evidence to assess its validity, results, and relevance before using it to inform a decision. This term is used for items 12 and 19 instead of “risk of bias” (which is more applicable to systematic reviews of interventions) to include and acknowledge the various sources of evidence that may be used in a scoping review (e.g., quantitative and/or qualitative research, expert opinion, and policy document). From: Tricco AC, Lillie E, Zarin W, O’Brien KK, Colquhoun H, Levac D et al. PRISMA Extension for scoping reviews (PRISMAScR): checklist and explanation. Ann Intern Med. 2018;169:467–473. doi: 10.7326/M18-0850.

## Appendix C

**Table A2:** Study characteristics.

Reference	Study Design	Sample	Study Protocol	Aerobic Exercise Intervention	Outcome Measures and Methods
Leddy et al., 2010 [36]	Prospective case series	N = 11 patients (age = 27.9 ± 14.3 years, women n = 4) with PCS (mean = 19 weeks post-concussion).	A GXT to determine symptom exacerbation was performed at baseline and 2 or 3 weeks after baseline. Following the second exercise test, participants were provided with an exercise prescription. The exercise intervention was performed until symptoms were no longer present. Telephone follow-up was completed 3 months following medical clearance.	5–6 days/week at 80% of the HR achieved at symptom exacerbation during the previous exercise test. Exercise duration was the same as that completed by the on their previous exercise test.	− Time to symptom exacerbation during exercise test. − HR via chest strap HR monitor. − PCSS via the graded symptom checklist.
Leddy et al., 2013 [24]	Pilot controlled trial	N = 12 with PCS (6–12 months post-concussion). -Exercise group, n = 4 (women n = 3). -Stretching (placebo) group, n = 4 (women n = 1). -Healthy controls, n = 4 (women n = 4).	At baseline, participants completed a math processing task under fMRI and a GXT before and after ~12 weeks of intervention. Participants underwent a 2nd fMRI once they could exercise to exhaustion without symptom onset/exacerbation. The healthy controls also underwent 2 fMRI exams at approximately the same time interval.	Exercise treatment was completed 6 days/wk for 20 min at 80% of the HR threshold determined with GXT. Stretching program included low-impact breathing and stretching for 20 min, 6 days/week that did not exceed 40–50% of age-predicted maximum HR.	− HR via Polar 810i T61 HR Monitor. − BP via sphygmomanometer. − PCSS via post-concussion scale. − Perceived exertion via Borg scale. − Cerebral blood flow was assessed via fMRI.
Maerlender et al., 2015 [26]	Pilot RCT	N = 28 college athletes. -Standard care n = 15, women n = 12. -Physical exertion group n = 13, women n = 8.	The initial clinical evaluation was performed by the athletic trainers. Sessions were discontinued for 24 h if symptoms occurred. Participants completed daily supervised AE until symptom resolution.	Athletes rode on a cycle ergometer for 20 min at RPE = 0–6 or until symptom exacerbation. Those in the non-intervention group were told to not do any physical activity exceeding normal activities required for school (i.e., walking to classes, studying, etc.).	− Daily physical activity via Actigraph. − PCSS and neurocognitive outcomes via the ImPACT test. − Perceived exertion via the Borg CR10 RPE scale.
Kurowski et al., 2017 [25]	Pilot RCT	N = 30; exercise group n = 15 (men n = 5, age = 15.2 ± 1.37 years). Stretching group n = 15 (men n = 8, age = 15.5 ± 1.8 years). Participants diagnosed with an mTBI and persistent symptoms for 4–16 weeks.	Participants were randomized into either a full-body stretching intervention or sub-symptom AE training intervention for 6 weeks. At baseline visit (week 0), participants underwent an aerobic bike test to determine the duration at which a symptom threshold occurred. There were two outcome assessments, one at baseline and another at week 7. Weekly assessments were performed to adjust the program at home as needed. Participants who returned to baseline at rest and were able to perform exercise program without onset of symptoms were considered to have recovered.	Those in sub-symptom AE training cycled 5–6 days/week for 80% of the duration that caused symptoms during the assessment visits. Participants in the stretching program were given a full-body stretch plan to complete 5–6 days/week. The stretching program rotated on a 2-week basis.	− Symptoms assessed via PCSI. − Physical activity log completed by patients.
Chrisman et al., 2017 [27]	Retrospective cohort study	N = 83 (14.9 ± 2.3 years of age), 54% women. 76% of concussions were due to sports. Participants had persistent concussion symptoms for greater than 1 month.	An initial physical evaluation and a Balke treadmill test to determine HR at symptom exacerbation were performed. Patients were provided with an at-home program to perform AE at 80% HR achieved on the Balke test. Symptoms were reported at appointments every 1–2 weeks until symptom-free.	Patients were instructed to perform physical activity at 80% of the HR achieved during exercise tolerance testing for the same duration that they could last during physical activity on the treadmill during the testing, or a maximum of 20 min.	− Concussive symptoms were reported and monitored via the SCAT-2.
Yuan et al., 2017 [39]	Secondary RCT analysis	N = 22 children (15.83 ± 1.77 years, women n = 10) with persisting symptoms (4–16 weeks post-mTBI) after mTBI. 17 completed the intervention, 8 in the aerobic exercise group, and 9 in the stretching group. 20 age- and sex-matched children were selected from an existing database for a healthy control comparison.	Adolescents diagnosed with an mTBI and having persistent PCS were randomized into either an AE or a stretching protocol group. They obtained MRI/diffusion tensor images as well as underwent symptom burden assessments. These were performed at initial study enrollment and again for 17/22 participants after 6–8 wks.	Used the same protocol as in Kurowski et al., 2017 [25].	− PCSI measured symptom burden. − Cognitive assessments were performed using the NIH toolbox cognitive batteries. − MRI and DTI were used to quantify structural connectivity and identify changes with AE.
McGeown et al., 2018 [28]	One group pre-test post-test pilot study	N = 9 (women n = 5) participants with persisting post-concussion symptoms (mean = 99.88 ± 79.95 days post-concussion) who were actively engaged in competitive sports. All were between the ages of 14 and 30 years old.	At the initial assessment, saliva was collected from patients to measure BDNF- concentrations. Patients completed the imPACT battery to test neurocognitive function. The PCSS testing was within the imPACT battery. Finally, patients completed the BESS protocol on a force platform to assess their static balance. After the initial assessment, patients received a structured exercise-based rehabilitation program, attending 12 sessions over 4 weeks. Following completion of the 12 sessions, participants completed a post-assessment identical to the initial assessment.	Patients attended 12 one-hour AEB training sessions over 4 weeks (3 sessions/week). Each session began with a warm-up followed by stationary cycling, static balance training, and finally cool-down exercises, completing 40–60 min sessions in total. HR intensity goal was calculated based on age-associated resting HR. Exercise intensity was initially 20% HR during week 1 and progressed to 50% HR during week 4.	− Resting HR was measured via the Polar FT2 HR Monitor worn by participants. − Neurocognitive function was assessed via the ImPACT battery. − Static balance via BESS. − Symptom burden was collected via PCSS scores within the ImPACT battery. − Salivary BDNF concentrations were determined by ELISA.
Micay et al., 2018 [20]	Pilot RCT	Adolescent men with an SRC who were symptomatic at PID 5 were randomized into the UC Group (n = 7, age = 15.6 ± 1 yrs) or the exercise group (n = 8, age = 15.8 ± 1.2 yrs).	Participants were assessed in weeks 1, 2, 3, and 4 post-injury on RTP progress and PCSS symptom severity. In addition to standard care, the exercise group performed a progressive exercise treatment until asymptomatic. PCSS scores were collected before and after each exercise session.	The AE intervention consisted of 8 sessions, each increasing in intensity. The first session was 10 min and at 50% HR max and progressed to 20 min at 70% age-predicted HR max for the final 3 sessions. Exercise occurred 2 days in a row then 1 day of rest, and this cycle was repeated for a total of 11 days.	− PCSS scores were used to evaluate symptom severity via SCAT 3.
Leddy et al., 2019 [17]	RCT	N = 103 adolescents with an SRC were assigned to either AE (n = 52, men = 46%, time since injury = 4.9 ± 2.2 days) or placebo-like stretching (n = 51, women = 47%, time since injury = 4.8 ± 2.4 days).	Participants were randomized to either stretching or AE during the acute phase after SRC. During the first visit, treadmill exercise tolerance was determined via BCTT. After initial visit, participants were followed up by their physician on a weekly basis until recovery or a maximum of 30 days. After 30 days, the participants who had delayed recovery were provided interdisciplinary treatment.	Both the AE and stretching interventions were performed for 20min/day. The AE group exercise at an HR target of 80% of the HR achieved at symptom exacerbation on BCTT. Participants were to stop their exercise if their symptoms increased 2 or more points from their pre-exercise symptom level or at 20 min if no symptoms arose. The stretching group followed a whole-body stretching protocol.	− Time between DOI and recovery (asymptomatic). Normal recovery was classified as <30 days and delayed was ≥30 days. − Exercise tolerance assessment was performed with the BCTT. − Participants reported symptoms using the PCS scale.
Leddy et al., 2019 [38]	Quasi-experimental design	N = 54; n = 24 in exercise group (15.13 ± 1.4 years old) and n = 30 in rest group (15.33 ± 1.4 years old). All participants were men with an acute SRC (<10 days post-concussion).	The rest group was from a previously published study and the exercise group was from an ongoing RCT. All exercise group participants completed the BCTT to determine exercise tolerance. The rest group was instructed to rest according to previously established standard of care. Both groups were followed up in clinic at day 7 and 14 after the initial visit. Participants used an online form to report their symptoms daily.	The exercise group was given a progressive program of 20 min or more of daily AE at an HR 80% of that they achieved during the BCTT. The rest group was instructed to not perform structured exercise.	− PCSS score was used to determine injury recovery. − HR was monitored via chest strap Polar H7 Bluetooth HR Sensory and Fitness Tracker.
Willer et al., 2019 [37]	Quasi-experimental design	N = 48, age = 15.4 ± 1 yrs, and women = 25% for rest group; n = 52, age 15.3 ± 2 yrs, and women = 46% for AE; n = 51, age = 15.4 ± 2, and women = 47% for placebo-like stretching. Participants were athletes with an SRC less than 10 days post-injury date.	The various groups were recruited at different time points and compared. All participants completed the BCTT to determine exercise tolerance and were assigned to their treatment group at the initial clinic visit and followed up by a physician weekly for the first 4 weeks, or until recovery. Symptom reporting took place on a password-protected online data form each evening between 7 and 10pm.	Participants in the exercise group were provided with a subthreshold AE prescription that was to be performed 20 min/day every day at 80% of the HR achieved at symptom exacerbation on the BCTT. Participants in the placebo group received a stretching plan which included breathing exercises and a guide book. They were instructed to stretch for 20 min/ day. The rest group was prescribed relative rest.	− Recovery was defined as symptom resolution to baseline and confirmation by a physician, as well as if they were able to exercise to exhaustion without worsening symptoms on the BCTT. − Symptom resolution was defined as a symptom severity score of less than or equal to 7 on the PCSS for 3 consecutive days.
Chrisman et al., 2019 [23]	Pilot RCT	N = 30 subjects completed the study. N = 11, age 15.8 ± 1.1 yrs, 54.6% women, and time since injury 75.9 ± 49.4 days in the stretching (placebo) group; n = 19, age 15.4 ± 1.8, 63.2% women, and time since injury 48.4 ± 32.2 days in the intervention group.	All participants completed in-person assessments at study entry including a modified BCTT (baseline) and 6 weeks post-intervention. The remaining assessments were completed online including weekly assessments of concussion symptoms. Other surveys were distributed at the 3- and 6-month follow-up. Accelerometer assessments were completed for 5–7 days at baseline and at 6 weeks to measure MVPA.	Placebo group was given an at-home stretching program to complete for 5–10 min daily. AE intervention was used for the 6-week daily home AE program. Duration of each daily exercise routine was 5–10 min/day greater than MVPA at baseline and increased weekly by 5–10 min/day via phone contact for a final goal of 60min/day. Intensity was 80% of HR at symptom exacerbation during the BCTT. AE type could be bike, treadmill, walking, stairs, or calisthenics.	− Feasibility was determined via enrollment, retention, engagement, and participant-reported satisfaction. − PCS was measured via the health behavior inventory at baseline, weekly, 3-month, and 6-month. − Quality of life was measured using the PEdsQL. − Fear avoidance of pain was measured via the Fear of Pain questionnaire.
Bailey et al., 2019 [22]	Pilot RCT	N = 16 (age = 15.75 ± 1.39 yrs, time post-injury = 56 ± 29.33 days, and women = 44% (n = 9, standard of care (control) and n = 7, AE) Concussions were both non-sport- and sport-related.	An initial evaluation of AE tolerance was performed and the daily exercise HR target was calculated. The intervention was 6 weeks long. Neuropsychological testing, postural stability testing, PCS-R, and BDI-2 were administered at baseline and following the intervention.	Daily exercise (3x 20 min intervals at HR 80% of identified threshold/week). The control group did 5 stretching activities daily for the first 3 weeks of the study, followed by 20 min of daily walking for the last 3 weeks.	− Symptom screening measures such as neuropsychological testing and postural stability testing were performed. − Concussion symptoms were measured via PCS-R. − Depressive symptoms via BDI 2.
Gladstone et al., 2019 [33]	Secondary outcome analysis RCT	N = 30; cycling group n = 15, age = 15.22 ± 1.37, time since injury = 52.3 ± 19.93 days, and men n = 5; Stretching (placebo) group, n = 15, age = 15.5 ± 1.8, and time since injury = 55.95 ± 22.16 days, men n = 8.	At baseline, all participants completed a graded aerobic bike test that increased in intensity until symptom exacerbation or a maximum of 30 min. Randomization occurred 1 week post-enrolment. Participants completed at least 6 weeks of training; if they did not return to baseline after 6 weeks, they remained for an additional 2 weeks prior to moving on to the post-intervention run-out period. Participants completed the NIH Toolbox and PedsQL at baseline and at the end of the intervention.	Participants randomized to the exercise treatment repeated the cycling test and were asked to exercise for 80% of the duration that exacerbated symptoms during the exercise test for 5–6 days/week. The exercise test was performed weekly and the exercise prescription was adjusted accordingly. Those in the stretching group had a full-body stretching program that was completed 5–6 days/ week. They received a new group of stretches every 2 weeks.	− Cognitive, sensory, motor, and emotional function was measured via the NIH Toolbox. − Quality of life was measured via PedsQL.
Snyder et al., 2021 [21]	Pilot RCT	N = 35 (non-injured controls, n = 10, women n = 4, and age = 20.4 ± 2 yrs; stretching and calisthenics (placebo), n = 13, women n = 7, age = 20.5 ± 2.8 yrs, and days since injury = 18.1 ± 3.3; AE intervention, n = 12, women n = 5, age = 22 ± 3.7 yrs, and time since injury = 20.1 ± 3.9 days).	Participants in the concussion group were instructed to avoid physical activity outside of the study during the 7-day intervention period. At pre- and post-intervention, participants completed the YMCA 3 min step test, SCAT3 was administered, the BDI, State-Trait Anxiety inventory, and a neuropsychological battery to assess attention, processing speed, memory, and executive functioning.	7-day, in-person exercise session administered by research staff with 1 rest day. This included a stationary bicycle at moderate intensity for 2 consecutive 20-minute periods with a 5-minute break in between. Including a 5 min warm-up and cool-down period. Moderate intensity was 65–75% of age-predicted maximum HR. A non-aerobic stretching/callisthenic movements program that did not exceed 50% of the participants’ age-predicted maximum HR was used for the placebo. Participants engaged in 2 consecutive 20-minute periods of non-AE with a 5-minute break led by trained research staff.	− Aerobic fitness at pre-intervention was measured via the YMCA 3 min step test. − Biopsychosocial function post-concussion was measured using a neuropsychological battery. − Symptom severity scores were measured from SCAT3. − Depression and anxiety were measured via the BDI and the State-Trait Anxiety inventory, respectively.
Howell et al., 2021 [34]	Cohort study	Participants undergoing the exercise intervention (N = 17) were 17.2 ± 2 years old. 41% of participants were women. Participants receiving standard of care (n = 20) were 16.8 ± 2.2 years and 50% were women. All participants were ≤ 14 days post-injury.	Participants completed an AE test (modified YMCA branching exercise test) within the first 14 days of injury. They were then randomized into the intervention group or standard of care group. They completed assessments at 1 month and 2 months after the initial visit. Participants recorded their exercise volume each week during the 8-week study period and logged their symptoms at each study visit (initial, 1 month, and 2 months).	The intervention group was instructed to exercise 5 days/ week for 20 min a day at a target HR of 80% of that achieved during the initial exercise test.	− Symptom severity was measured using the PCSI. − Exercise volume was self-reported using REDcap.
Chrisman et al., 2021 [19]	Pilot clinical trial	N = 19 (79% women) with an average age of 14.3. Participants had a mean duration of symptoms of 75.6 days.	All youth enrolled received a 6-week MSTEP intervention which involved wearing a Fitbit and weekly HR/ exercise duration goals. Participants met over Zoom with a research assistant weekly. Online surveys were completed at baseline, 3 weeks, and 6 weeks.	At-home AE program was performed daily for 6 weeks. The initial goal was set for 10 min at an HR of 120 bpm. Type of AE was up to the individual, but if symptoms got worse during exercise, they were told to take a break and decrease the HR goal until they were able to tolerate 10 min of exercise. Weekly goals progressed until a max of 60 min of physical activity/day at an HR of 140 was reached.	− HBI was used to assess concussive symptoms. − Fear of Pain questionnaire was used to assess fear avoidance. − PedsQL was used to assess health-related quality of life. − Feasibility and satisfaction with the exercise program was measured via the self-reported Satisfaction with Study questionnaire. − Depressive symptoms were measured via the Patient health Questionnaire-9. − Anxiety symptoms were measured using the GAD-7. − Sleep quality was measured via Adolescent Sleep–Wake Scale-10.
Leddy et al., 2021 [15]	RCT	N = 118 adolescents with SRC; exercise group, n = 61, 38% women, age = 15.5 ± 1.4 yrs, and time post-injury = 5.8 ± 2.3 days; stretching group, n = 57, 37% women, age = 15.9 ± 1.4 yrs, and time post-injury = 6.3 ± 2.4 days.	Participants were randomized to either AE or stretching (control) interventions for up to 4 weeks at 3 sport medicine clinics. Every week, participants completed a BCTT and medical exam until they are recovered or upon reaching 4 weeks.	The AE group completed 20 min of AE at home each day at HR up to 90% of the maximum achieved during their BCTT. The stretching group performed light stretches and breathing exercise that would not increase their HR.	− Symptoms severity was assessed with the PCSI and included in the determination of clinical recovery.
Hutchison et al., 2022 [16]	Longitudinal randomized control trial	N = 38 participants (SAEP group, n = 20, age mean = 18 yrs, range = 16–19 yrs, women = 65%; standard care group, n = 19). Age mean = 21 range = 16–22, women = 53%) with an acute SRC.	Participants were randomized to either SAEP or UC exercise protocol at the initial visit. Participants were seen by a physician for follow-up across 28 days and completed follow-up with a member of the research team on days 7, 14, 21, and 28. At each visit, participants gave a symptom severity rating.	UC exercise protocol had a brief period of rest (cognitive and physical) before gradually increasing sub-symptom exacerbation exercise. The SAEP group began exercise at PID 3. This protocol consisted of 8, 20 min sessions over 11 days. 2 consecutive days of exercise were followed by 1 day of rest. Protocol progressed from 25 min to 30 min and from 60% to 75% age-predicted maximum HR at each session through the 11-day period.	− Days to asymptomatic status and days to medical clearance were the primary outcome measures. − Symptom severity was measured via the SCAT-5.
Howell et al., 2022 [18]	Pilot RCT	N = 17(intervention, n = 9, age = 14.2 ± 2.1 yrs, women = 56%, and time post-injury = 5.5 ± 1.2 days; standard care, n = 7, age = 13.6 ± 1.7, women = 57%, andtime post-injury = 5.6 ± 1.8). Patients were ≤ 7 days post-concussion and obtained a “predicting/preventing post-concussive problems in pediatrics” (5P) risk score of 6 or greater.	Prior to randomization, all participants completed a modified version of the YMCA branching exercise test. Patients completed a VAS to describe their current symptom severity prior to the test. Patients were then randomized to early AE treatment or standard care for 30 days. The PCSI was completed at study initiation and at study completion.	The early AE group exercised at 80% of the HR achieved during the YMCA bike test for 20 min each day, 5 days/week. The standard care group were told to gradually progress physical activity that did not exceed symptom exacerbation thresholds.	− PPCS scores were measured via PCSI. − -Physical activity participation was measured via Wrist-Actigraph data.
Chizuk et al., 2022 [32]	Secondary analysis of RCT	N = 51; adherent group n = 31, age = 15.51 ± 1.5, men = 57%, and time since injury = 6.24 ± 2.4 days; non-adherent group, n = 20, age = 16.18 ± 1.7, men = 63%, and time since injury = 6.84 ± 1.8 days.	Participants were randomized to an individualized AE program or a placebo stretching program. In this secondary analysis, only participants in the AE group were included. All participants completed the PCSI daily. Weekly BCTTs (for 4 weeks or until recovered) were completed to adjust the target HR for the exercise intervention.	Participants were to perform at least 20 min of AE of their choice (walking, jogging, biking, or stationary biking) daily for 6 out of 7 days at 90% of the HR threshold which caused symptoms on the previous BCTT.	− Recovery was defined as resolution of symptoms, normal physical examination, and ability to exercise to at least 80% of age-appropriate max HR without symptom onset on the BCTT. − Symptom severity was measured via PCSI.
D’Alonzo et al., 2023 [31]	Secondary analysis of an RCT	N = 54 adolescents with an SRC (median age = 15.8, median time from injury = 6 days, men = 61.8%) were randomized into aerobic or stretching exercise groups.	Following an initial appointment and completion of the BCTT, participants were randomized into prescribed sub-symptom threshold AE or stretching exercise groups. The intervention lasted 4 weeks and included weekly visits and a 4-month post-injury follow-up. Concussion symptoms were collected using PCSI 3 times/day.	In the AE group, participants were asked to exercise up to 90% of the HR achieved during their previous BCTT for 20 min each day. New HR targets were calculated each week with reassessment of their exercise tolerance during a BCTT.	− Time to return to pre-injury symptoms and exercise tolerance were the primary outcomes. − PPCS severity was measured using PCSI. − Patient anxiety symptoms were measured via PROMIS Anxiety pediatric short form score.
Mercier et al., 2024 [35]	Prospective cohort study	N = 50, (diagnosis of PPCS and exercise intolerance); i -AEP group (n = 27, age = 43.5 ± 10.5yrs); delayed-AEP group (n = 23, age = 41.5 ± 11.2 yrs). No reported sample size for men or women.	Participants were randomized into the i-AEP group or the d-AEP group. i-AEP group completed a 12-week AE intervention. The d-AEP group completed a 6-week stretching intervention prior to engaging in the AE program. Both groups completed a BCTT test before beginning the AE program and every 3 weeks to update AE prescription. Questionnaires were all completed at study initiation, 6 weeks, and 12 weeks.	The AEP consisted of sub-symptom threshold AE at an HR of 70–80% max HR achieved on the BCTT completed 5d/week for 30 min. The d-AEP group completed a 6-week stretching intervention followed by 12 weeks of sub-symptom threshold AE. The stretching consisted of 30 min performed 5d/week at an HR that did not exceed 50% of age-predicted max HR (220-age).	− Concussion symptoms were evaluated via RPQ. − QoL was determined via QOLIBRI. − Depressive symptoms were evaluated via PHQ-9, − Anxiety symptoms were evaluated via GAD-7, − The functional impact of headaches was evaluated via HIT-6, − Fatigue was measured via FSS. − Daytime sleepiness was evaluated via ESS, − Dizziness was evaluated via DHI.
Mercier et al., 2025 [14]	RCT	N = 52, months post-mTBI = 24.7 ± 14.0; AE group, n = 27 (women, n = 18) and age = 43.5 ± 10.5 yrs; placebo (stretching) group, n = 25 (women, n = 21) and age = 42.6 ± 11.6 yrs.	Participants were randomized to sub-symptom threshold AE or placebo (stretching). A BCTT was completed at baseline and every 3 weeks to update HR prescription for those in the AE group. Participants completed PROM at baseline and following the 6-week intervention.	Participants randomized to the AE group performed 30 min of sub-symptom threshold AE 5d/week at 70–80% of the maximum HR achieved during the previous BCTT. The Stretching group was prescribed low-intensity stretching to perform 30 min/d, 5d/week at an HR that does not exceed 50% of their age-predicted HR max.	− Symptom burden was determined via RPQ. − Adherence to exercise was tracked via exercise diary. − QoL was evaluated via QOLIBRI questionnaire. − Depressive symptoms were assessed via PHQ-9. − Anxiety symptoms were assessed via GAD-7. − The functional impact of headache was assessed via HIT-6. − Fatigue was assessed via FSS. − Daytime sleepiness was assessed via ESS. − Dizziness was assessed via DHI. Sleep was assessed using via wrist-worn accelerometer and a sleep diary.
Thomas et al., 2025 [13]	Multicenter, prospective RCT	N = 235 (women n = 114; men n = 121; age 14.5 ± 2.3) with an acute mTBI (≤72 hrs post-injury).	Baseline assessments for demographics, injury details, symptoms, PA, and QoL. Participants were randomized into 4 different groups: UC, EA, UC plus mHealth, or EA plus mHealth. Participants were provided with activity trackers and an active injury management app to track symptoms, cognitive activity, and compliance. Follow-ups were at 3–5 days, 14 days, 1 month, and 2 months.	The EA group was instructed to perform light-to-moderate PA and to achieve 10,000 steps, progressing in the number of days they reach the target each week. The UC group engaged in symptom-guided return to activity following 48 h of rest.	− PA compliance was monitored with a Fitbit activity monitor. − Symptom severity was evaluated via PCSS. − QoL was evaluated via PedsQL, − Neurocognitive performance was assessed via ImPACT quick test. − Balance was assessed using BESS. − Anxiety- and depression-specific symptoms were assessed via BSI-18. − Somatic symptoms were captured with the Somatization Subscale of Child Behavior Checklist. − Vestibular–oculomotor function was assessed via VOMS.
Bogdanowicz et al., 2025 [30]	Secondary analysis of an RCT	N = 111; aerobic exercise group n = 56 (men n = 36, women n = 20, age = 15.26 yrs (95% CI = 14.8, 15.94), median days post-concussion = 5); stretching group n = 55 (men n = 35, women n = 20, age = 16.14 yrs (95% CI = 15.45, 16.68) median days post-concussion = 6.	At the initial appointment, patients underwent a physical exam, completed a PedsQL questionnaire and PCSI, and underwent a BCTT. Participants were randomly assigned to the AE or stretching treatments. A BCTT was completed weekly for a maximum of 4 weeks, at which the PedsQL questionnaire was completed again. The stretching group was instructed to perform stretches and breathing exercises at a light intensity. The intervention continued until the patient recovered.	The AE group were instructed to perform AE at an intensity equivalent to 90% of the HR achieved during the exercise tolerance test for a minimum of 20min/day.	− Quality of life was assessed via PedsQL questionnaire. − Symptom severity was determined via PCSI.
Castellana et al., 2025 [29]	Exploratory secondary analysis of 2 RCTs	N = 198; aerobic exercise group, n = 102 (men, n = 59), age = 15.37 ± 1.57 yrs, days post-injury = 5.42 ± 2.30 days; stretching group, n = 96 (men, n = 58), age = 15.75 ± 1.60 yrs, days post-injury = 5.71 ± 2.47 days.	Patients were given either a PCSI or PCSS to assess symptoms at initial visit. Patients completed a BCTT to assess exercise intolerance and were randomized into AE or placebo (stretching). BCTT was performed weekly to update target HR for the AE group. Each group was stratified into low- and high-mood symptom burden for analysis.	Patients in the AE group were instructed to exercise between 80 and 90% of the target HR they achieved on the BCTT for a minimum of 20 min/day. Exercise was to be stopped if concussion symptoms increased to more than mild.	− Symptom severity was determined via PSCI or PCSS.

PCSS, Post-Concussion Symptom Scale; SRC, sport-related concussion; ImPACT, Immediate Post-concussion and Cognitive Test; DOI, Day of Injury; PID, Post-Injury Day; PCS, Post-Concussion Symptoms; HR, heart rate; BDI, Beck Depression Inventory; fMRI, functional magnetic resonance imaging; MRI, Magnetic Resonance Imaging; RPE, rated perceived exertion; PCSI, Post-concussion symptom inventory; SCAT 2, 3, 5, Sport Concussion Assessment Tool-2, 3, 5; AEB, aerobic and balance exercise; BCTT, Buffalo Concussion Treadmill Test; MVPA, Moderate and Vigorous Physical Activity; PCS-R, Post-concussive scale-revised; PPCS, persistent post-concussive symptoms; mTBI, mild traumatic brain injury; SAEP, structured aerobic exercise protocol; VAS, visual analog scale; AE, aerobic exercise; UC, Usual Care; BP, Blood Pressure; RCT, Randomized Controlled Trial; GXT, Graded Exercise Stress Test; NIH Toolbox, National Institute of Health Toolbox; DTI, Diffusion Tensor Imaging; BDNF, Brain-Derived Neurotrophic Factor; BESS, Balance Error Scoring System; ELISA, Enzyme-Linked Immunoassay; PEDsQL, Pediatric Quality of Life Inventory; MSTEP, Mobile Subthreshold Exercise Program; GAD-7, Generalized Anxiety Disorder Scale-7; i-AEP, immediate-aerobic exercise protocol; d-AEP delayed aerobic exercise following stretching protocol; RPQ, Rivermead Post-Concussion Symptoms questionnaire; QOLIBRI, Quality of Life After Brain Injury questionnaire; PHQ-9, Patient Health Questionnaire; HIT-6, Headache Impact Test; FSS, Fatigue Severity Scale; ESS, Epworth Sleepiness Scale; DHI, Dizziness Handicap Index; AEP, aerobic exercise protocol; PA; physical activity; PROMIS, Patient-Reported Outcomes Measurement Information System; HBI, health behavior inventory; PROM, Patient-Reported Outcome Measure; QoL, quality of life; EA, Early Activity; BSI-18, brief symptom inventory; VOMS; Vestibular–Ocular Motor Screening.

**Table A3:** Study results and conclusions.

Reference	Measured Patient-Reported Outcomes	Relevant Main Results	Conclusion
Leddy et al., 2010 [36]	PCS symptoms	− Athletes recovered faster than non-athletes (25 ± 8.7 vs. 74.8 ± 27.2 days, *p* = 0.01). − Significant reduction in overall mean symptom score between baseline and treatment periods (*p* = 0.002).	Prescribed exercise treatment for management of patients with PCS results in improved patient-reported symptoms when compared with a no-treatment baseline.
Leddy et al., 2013 [24]	PCS symptoms.	− Exercise treatment resulted in a decrease in the number of symptoms (*p* < 0.0004), while no change was found with the stretching condition.	Exercise treatment could improve the number of symptoms and may help restore cerebral blood flow regulation in individuals with PCS.
Maerlender et al., 2015 [26]	ImPACT symptom scores.	− The median number of days to symptom resolution and recovery was no different between exercise and standard care groups, *p* = 0.705.	An AE intervention utilizing moderate physical activity did not improve symptom resolution or change time to recovery compared to UC.
Kurowski et al., 2017 [25]	PCSI	− There was greater improvement in the exercise group than in the stretching group, *p* = 0.044 in PCSI scores.	Findings suggest that the sub-symptom exacerbation aerobic training has potential to be beneficial for teens with persistent PCS after an mTBI.
Chrisman et al., 2017 [27]	Self-reported symptom questionnaires	− Most patients improved following the intervention with no reports of worsening symptoms. − Symptoms decreased exponentially after starting the SSTEP and this did not differ by time post-injury at presentation (i.e., <6 weeks, 6–12 weeks, >12 weeks) (*p* = 0.006).	Concussion symptoms decreased exponentially after the addition of monitored exercise intervention. The decrease rate was similar for youth at various time points from injury (a few weeks to several months).
Yuan et al., 2017 [39]	PCSI	− After intervention, there was an improvement in PCSI, which was correlated with change global efficiency with aerobic exercise therapy (*p* = 0.046). Change in path length was also correlated with change in PCSI in the aerobic training and stretching comparison group (*p* < 0.05).	This study shows that changes in PCSI following aerobic exercise therapy are associated with changes in brain network connectivity in children with PPCS after mTBI.
McGeown et al., 2018 [28]	Changes in symptom scores	− There was a reduction in PCSS scores (*p* < 0.01) following treatment.	These findings suggest that AEB treatment can improve PCS scores in addition to improvements in visual motor speed and static balance.
Micay et al., 2018 [20]	PCSS	− AE group experienced greater symptom resolution compared to the UC group over the recovery timeline (AE group, 15.8 ± 1.9 vs. 6.7 ± 4.1 points vs. UC group, 18.8 + 4.9 points vs. 10.0 ± 6.1 points)	Results indicate that a structured AE program is safe for the post-acute stage of SRC in adolescents, and due to the increased symptom resolution compared to UC it should be explored as a full phase 3 clinical trial.
Leddy et al., 2019 [17]	PCS	− AE participants recovered in a median of 13 days, compared to 17 days (*p* = 0.009) for the stretching group. − More participants in the stretching group (n = 7) experienced delayed recoverycompared to the exercise group (n = 2) (*p* = 0.08) − Total symptom scores decrease more rapidly with exercise, however this was not statistically significant.	Individualized sub-symptom threshold AE treatment prescribed during the acute phase after SRC safely speeds recovery in adolescents. The study provided preliminary evidence that subthreshold exercise treatment can reduce incidence of delayed recovery.
Leddy et al., 2019 [38]	Days to recovery, PCSS	− The exercise group had a shorter recovery time (8.29 ± 3.9 days) compared to the rest group (23.93 ± 41.7 days; *p* = 0.048). − No participants in the exercise group experienced delayed recovery (>30 days), while 4/30 of the rest group had delayed recovery. − On day 14, fewer participants in the exercise group remained symptomatic compared to the rest group (*p* = 0.028).	The results of this study suggest that early subthreshold AE prescribed to adolescent men with acute SRC speeds up recovery and has the potential to prevent delayed recovery.
Willer et al., 2019 [37]	SCAT 3, PCSS	− Rest group recovered in 16 days, which was delayed compared to the exercise group who recovered in 13 days (*p* = 0.020). − The placebo group recovered in 17 days.	Rest and placebo-like stretching was similar in terms of days to recovery and symptom recovery pattern for SRC, however both were less effective than a sub-symptom threshold AE prescription in symptom resolution.
Chrisman et al., 2019 [23]	Trajectory of concussive symptoms (HBI), Pediatric Quality of Life inventory, Physical activity changes, Fear of Pain questionnaire	− Concussion symptoms decreased quicker in the intervention group compared to control (*p* = 0.02). − Health-related quality of life improved over time; this was not different by groups.	This study suggests the feasibility and benefit of a 6-week subthreshold exercise program with few in-person clinic visits for adolescents with persistent SRC.
Bailey et al., 2019 [22]	BDI-2, PCS-R	− For all groups a 60% improvement in concussion symptoms was noted. − Removing depression, the exercise program had a large effect on symptom reduction (*p* < 0.05) compared to the control group (UC).	Results indicate that exercise is effective in reducing symptoms in youth with persistent concussion symptoms.
Gladstone et al., 2019 [33]	PedsQL and NIH Toolbox	− There was no difference between groups.	Results showed improvement in both aerobic and stretching groups in QoL, but results suggest that only aerobic training would be beneficial for neurocognitive recovery based on the NIH Toolbox.
Snyder et al., 2021 [21]	SCAT3, BDI-2, Sleep Problem Index-II	− Symptom severity scores decreased from pre- to post-intervention for both groups (*p* = 0.007), with no difference between groups (*p* = 0.78). − BDI-2, Sleep Problem Index-II, and BESS improved post-intervention but no differences in trajectory of improvements between groups (*p* = 0.21, 0.09, and 0.14, respectively).	Results suggest that AE does not negatively impact recovery trajectories, but tolerability may be lowered for patients with high symptom scores.
Howell et al., 2021 [34]	PCSI	− There was no difference between the intervention group and UC group at the initial (*p* = 0.26), 1-month (*p* = 0.96) or 2-month (*p* = 0.11) timepoints. − Exercising less than 100 min/week resulted in higher symptom severity scores at 1-month compared to those who exercised 100 min or more each week (*p* = 0.03).	There was no difference in symptom burden between the intervention and standard care group. However, higher exercise volume was associated with decreased symptom burden.
Chrisman et al., 2021 [19]	HBI, PedsQL, Fear of Pain questionnaire, Symptoms of anxiety (GAD7), Symptoms of Depression (PHQ9), Adolescent Sleep–Wake Scale	− Concussive symptoms (*p* = 0.0002) and fear avoidance decrease (*p* < 0.0001), while PedsQL improved (*p* < 0.0001) over the 6 wk intervention. − Symptoms of anxiety (*p* = 0.0003) and depression improved (*p* < 0.0001), so as sleep (*p* < 0.0001).	The results of this study suggest that telehealth delivered subthreshold exercise programs can improve PROMs in adolescents with persisting concussion symptoms.
Leddy et al., 2021 [15]	PCSI assessed daily	− AE resulted in decreased time to clinical recovery (i.e., return of symptoms to pre-injury levels; median 14 days) compared to the stretching group (median 19 days) (*p* = 0.039).	Sub-symptom threshold AE results in the recovery of patient-reported symptom severity more quickly than the control (stretching) group.
Hutchison et al., 2022 [16]	Symptom severity score	− The SAEP group were asymptomatic faster and had a lower symptom severity score at each 1-week assessment.	The findings of this study suggest that SAEP can improve the rate of symptom resolution.
Howell et al., 2022 [18]	PPCS	− A smaller proportion of the intervention group (44%) developed PPCS when compared to the standard of care group (86%; relative risk = 0.52; 95% CI = 0.34–1.36). − There was no difference in symptom severity 1-month post-concussion between groups (*p* > 0.05).	Study results suggest that early AE may lower the risk of PPCS compared to standard of care.
Chizuk et al., 2022 [32]	PCSI	− Those more adherent to exercise program recovered faster than those who were not (22 days vs. 21.5 days *p* = 0.016). − Adherence during week 1 was inversely correlated to recovery time (*p* = 0.046).	The results of the study suggest that adherence to AE within the first week of concussion is associated with faster recovery. Data suggests that initial degree of exercise tolerance can impact adherence to AE.
D’Alonzo et al., 2023 [31]	PROMIS anxiety score, PCSI	− No difference in median time to symptom resolution between the low-PROMIS anxiety and the the high-PROMIS anxiety group (low-PROMIS = 10 days vs. high-PROMIS = 12 days; *p* = 0.62). − Each additional unit of PROMIS anxiety score was associated with a 1.52-unit higher PCSI score (*p* < 0.001). − Neither effect varied between AE/stretching group.	The results of this study show that a higher initial PROMIS anxiety score was not associated with delayed time to symptom resolution. But the PROMIS anxiety score was associated with an increased PCSI score regardless of exercise group.
Mercier et al., 2024 [35]	RPQ, QOLIBRI, PHQ-9, GAD-7, HIT-6, FSS, ESS, DHI	− i-AEP resulted in improved RPQ (*p* < 0.001), QOLIBRI (*p* < 0.001), PHQ-9 (*p* = 0.001), HIT-6 (*p* < 0.001), FSS (*p* < 0.001), and DHI (*p* = 0.001). − Compared to the d-AEP group, i-AEP had greater changes in RPQ (*p* = 0.042), HIT-6 (*p* = 0.002), and FSS (*p* = 0.013).	− A 12-week AEP initiated immediately after a concussion can improve symptom burden, QOL, depressive symptoms, functional impact of headaches, fatigue, and dizziness. Initiating an AEP early after concussion may increase the change in symptom burden, functional impact of headaches, and fatigue to a greater extent than delaying the AEP.
Mercier et al., 2025 [14]	RPQ, QOLIBRI, PHQ-9, GAD-7, HIT-6, FSS, ESS, DHI	− The AE intervention resulted in improved symptom burden (*p* < 0.001), QOLIBRI (*p* < 0.001), depressive symptoms (*p* = 0.025), anxiety symptoms (*p* = 0.021), functional impact of headache (*p* = 0.002), and fatigue (*p* = 0.004). − Per-protocol analysis identified QOLIBRI improved to a greater extent with AE compared to placebo (*p* = 0.047).	This study provides evidence to support the prescription of sub-symptom threshold AE for adults with PPCS. AE is associated with a greater improvement in QoL compared to stretching following a 6-week intervention.
Bogdanowicz et al., 2025 [30]	PedsQL, PCSI	− AE decreased recovery time (*p* = 0.043) compared to the stretching condition, but there was no difference in the change in PedsQL between the groups.	AE decreased time to recovery but did not improve QoL more than stretching at the 4-week time point.
Thomas et al., 2025 [13]	PCSS, BSI-18	− At day 5, participants prescribed AE reported a greater number of symptoms (*p* < 0.05), but this difference was not present at days 14, 30, or 60. − The group performing AE took a median of 10 days for symptom resolution compared to 5 days for the usual care group (*p* < 0.03). − There were no differences in depression or anxiety symptoms between groups.	Early prescribed AE may worsen symptom burden immediately following an mTBI and increase the time to recovery compared to usual care. Early AE does not change patient reporting of anxiety- or depression-related symptoms.
Castellana et al., 2025 [29]	PCSS	− Individuals with a lower-mood symptom burden recovered more quickly than individuals with a higher-mood symptom burden regardless of intervention (21.58 ± 30.67 days vs. 42.03 ± 51.86 days, respectively (*p* = 0.023). − The incidence of PPCS was lower in the low-mood symptom burden compared to the high-mood symptom burden group (*p* < 0.001). − Participants with a high-initial-mood symptom burden who performed AE recovered quicker than those in the placebo group (*p* = 0.005).	Engaging in early AE reduced the incidence of PPCS in adolescents with high-mood symptom burden.

PCSS, Post-Concussion Symptom Scale; SRC, sport-related concussion; ImPACT, Immediate Post-concussion and Cognitive Test; PCS, Post-Concussion Symptoms; BDI, Beck Depression Inventory; PCSI, Post-concussion symptom inventory; SCAT 2, 3, 5, Sport Concussion Assessment Tool-2, 3, 5; PCS-R, Post-concussive scale-revised; PPCS, persistent post-concussive symptoms; mTBI, mild traumatic brain injury; SAEP, structured aerobic exercise protocol; AE, aerobic exercise; UC, Usual Care; NIH Toolbox, National Institute of Health Toolbox; PEDsQL, Pediatric Quality of Life Inventory; GAD-7, Generalized Anxiety Disorder Scale-7; i-AEP, immediate-aerobic exercise protocol; d-AEP delayed aerobic exercise following stretching protocol; RPQ, Rivermead Post-Concussion Symptoms questionnaire; QOLIBRI, Quality of Life After Brain Injury questionnaire; PHQ-9, Patient Health Questionnaire; HIT-6, Headache Impact Test; FSS, Fatigue Severity Scale; ESS, Epworth Sleepiness Scale; DHI, Dizziness Handicap Index; AEP, aerobic exercise protocol; PROMIS, Patient-Reported Outcomes Measurement Information System; HBI, health behavior inventory; PROM, Patient-Reported Outcome Measure; QoL, quality of life; EA, Early Activity; BSI-18, brief symptom inventory.
